# Referral to an Electronic Screening and Brief Alcohol Intervention in Primary Health Care in Sweden: Impact of Staff Referral to the Computer

**DOI:** 10.1155/2011/918763

**Published:** 2011-04-13

**Authors:** Preben Bendtsen, Diana Stark Ekman, AnneLie Johansson, Siw Carlfjord, Agneta Andersson, Matti Leijon, Kjell Johansson, Per Nilsen

**Affiliations:** ^1^Division of Community Medicine, Department of Medical and Health Science, Linköping University, 58381 Linköping, Sweden; ^2^Department of Medicine (MSK), Motala Hospital, 591 85 Motala, Sweden; ^3^Department of Public Health Science, Faculty of Social and Life Sciences, Karlstad University, 651 88 Karlstad, Sweden; ^4^Department of Health and Culture, University West, 46186 Trollhättan, Sweden; ^5^R&D Department of Local Health Care, County Council of Östergötland, Linköping University, 582 85 Linköping, Sweden

## Abstract

The aim of this paper was to evaluate whether primary health care staff's referral of patients to perform an electronic screening and brief intervention (e-SBI) for alcohol use had a greater impact on change in alcohol consumption after 3 month, compared to patients who performed the test on their own initiative. Staff-referred responders reported reduced weekly alcohol consumption with an average decrease of 8.4 grams. In contrast, self-referred responders reported an average increase in weekly alcohol consumption of 2.4 grams. Staff-referred responders reported a 49% reduction of average number of heavy episodic drinking (HED) occasions per month. The corresponding reduction for self-referred responders was 62%. The differences between staff- and self-referred patient groups in the number who moved from risky drinking to nonrisky drinking at the followup were not statistically significant. Our results indicate that standalone computers with touchscreens that provide e-SBIs for risky drinking have the same effect on drinking behaviour in both staff-referred patients and self-referred patients.

## 1. Introduction

More than two decades ago, the World Health Organisation (WHO) suggested that screening and brief intervention (SBI) for risky alcohol consumption, including hazardous and harmful alcohol consumption but excluding alcohol misuse/dependence, should be integrated in the daily routine of primary health care (PHC) in order to provide early intervention for nontreatment-seeking, nonalcohol-dependent drinkers. Since then, numerous research projects have been carried out in order to establish the scientific evidence for various methods of delivering brief alcohol interventions in PHC and other health care settings [1]. The scientific literature has provided evidence that supports the efficacy of SBI as studied in RCT studies, where mostly research staff has performed the SBI to ensure a consistency in the content of the SBI. Fewer studies have examined the effectiveness of SBI with ordinary staff performing the interventions; however, the distinction between efficacy and effectiveness studies is not always clarified. So far, 15 systematic reviews and meta-analyses of SBI study have been publishing data since 1993, the most recent being a comprehensive 2007 Cochrane review by Kaner et al. [2].

Despite convincing evidence for the efficacy and, to a certain extent, the effectiveness of SBI, the uptake of such interventions in routine health care has been slow. Research has identified several barriers to more widespread implementation and use of these interventions. Due to the perceived sensitivity of the alcohol issues, health care professionals generally find it difficult to raise the issue of alcohol drinking with patients who are not seeking help for alcohol-related problems. Many are afraid of provoking negative reactions and losing rapport with patients. Most health professionals have received little or no training in addressing alcohol use, either in their undergraduate education or continuing professional education, and they do not feel confident in their abilities to intervene with alcohol problems [1]. Moreover, with regard to situational and contextual factors, a common finding is that perceived lack of time for overburdened health professionals constitutes a considerable barrier for addressing alcohol issues [1, 3]. 

Computerized alcohol intervention approaches have shown advantages over face-to-face counselling in terms of potentially being more easily implemented into health care settings, thus overcoming some of the identified obstacles concerning regular alcohol interventions [4–9]. Electronic SBI, or e-SBI, can be provided for the general public via Internet-based programmes or target specific groups of people who have, or suspect they have, health risks. In a recent review on the impact of Internet behaviour change interventions including 85 studies (with a total sample of 43,236 participants), a variety of e-SBIs showed a small but significant effect on various health behaviours. Population health effects of e-SBI may be considerable since this form of intervention has the potential to reach a large number of individuals [10]. 

However, only a few studies have studied computerized technologies used on site in PHC and how this technology may integrate with the existing daily routine. Such a computerized health behaviour intervention could have the same content as an internet-based intervention, linking to webpage or a local application. This mode of delivery may offer a good complement to Internet-based health behaviour interventions due to the high proportion of individuals seeking PHC [11]. e-SBI approaches offered on site in a primary health care setting to specifically address alcohol use have predominantly been used with young adults attending university or college student health care [12–15]. More recently, studies have emerged that evaluate the effectiveness of e-SBI in adult patients as part of the daily routine in emergency care [5, 8, 9, 13, 16]. 

Despite the promising results shown from interventions by electronic media, little has been published describing effectiveness of e-SBIs offered as part of a routine on site in general PHC populations under naturalistic conditions, and whether such interventions should be a free access for patients seeking primary health care or if they should be restricted to patients who are referred by staff to perform the test. 

The aim of this study was to evaluate whether an active request from primary health care staff to perform an e-SBI had a greater impact on change in alcohol consumption after 3 months compared to patients who performed the test on their own initiative.

## 2. Methods

### 2.1. Study Location and Population

The study location was set in Östergötland County, Sweden. The county's population during the study period was approximately 420,000 inhabitants. These inhabitants are considered representative of the larger Swedish population in terms of age distribution, employment rates, and economy, and they come from a mix of rural and urban communities. There are 42 PHC units operating within the county. The 42 units differ with regard to the number of general practitioners (GPs), nurses, and other staff members employed. The units are situated in both urban and rural areas. Swedish health care is publicly funded, that is, residents are insured by the state, and health care services are funded through a taxation scheme of the county councils. The number of PHC units offering patients the e-SBI was successively extended as part of an ongoing implementation project during the study period, from 10 units in 2007 to 28 units in 2009. All e-SBIs were performed anonymously as part of the routine health care services. Patients were consecutively recruited into the study during a two-year period, from September 2007 to August 2009.

### 2.2. The Computerized Concept

The PHC units that were included in the study were equipped with a set of one or two computers, monitors, and printers, depending on enrolled patient population size, all included in stand-alone, touchscreen IT kiosks. The e-SBI programme, including screening questions and brief interventions, was developed by the Lifestyle Intervention Research Group at Linköping University. It was based on previous findings from Swedish-language e-SBIs set in student health care and emergency department settings [5, 6, 12–14, 16]. The e-SBI included health-related questions regarding alcohol consumption and physical activity, motivation to change, and attitudes to performing the test. All respondents receive personalized written feedback printed out at the kiosk after completing their tests. For this paper, only the alcohol-related data were analyzed. 

Weekly alcohol consumption was measured based on beverage-specific self-report of day-by-day consumption during a typical week in the last 3 months, measured by standard drinks, defined in Sweden as 12 grams of alcohol. Risky drinking in this study was defined for women as 10 or more standard drinks per week and/or 4 standard drinks per occasion (heavy episodic drinking (HED) at least once a month. For men, the definition of risky drinking was 15 or more standard drinks per week and/or 5 standard drinks per occasion (HED) once a month or more often. These are the suggested risky drinking limits in Sweden, as defined by the National Institute of Public Health. 

The individual patient's alcohol consumption was compared with the suggested sensible limits in Sweden followed by a short tailored advice based on the patient's actual consumption. However, patients who exceeded the risky drinking levels for HED (i.e., had more than 4 standard drinks on one occasion for females, and more than 5 standard drinks on one occasion for males) one to three times a month were informed that they were drinking an amount of alcohol that “increases the risk” for adverse effects, and when they consumed this amount once a week or more often they were informed that they drank at “risky levels.” Concerning the average weekly consumption in the written feedback, the patients were informed that they were drinking on a risky level when consuming above the national risky drinking levels, and they were also told that drinking between 1 or 3 drinks under these limits still incurred an increased risk for negative consequences. If the respondent reported no alcohol consumption during the last 3 months, the subsequent questions about alcohol use in the e-SBI were omitted. 

Motivation to change was assessed in the computerized test, making it possible to provide feedback on the patients' consumption with reference to their motivation to change. For example, if a patient's drinking was risky and he or she was not motivated to change, the feedback did not suggest the patient to decrease the drinking, instead giving more of a reflective feedback on the answers, such as, “your consumption is at a risky level, but you do not appear to have any intention to change your drinking.” The feedback was one page for alcohol and one page for physical activity.

### 2.3. Study Procedure

The data for this study were obtained from a convenience sample of patients visiting PHC as part of routine primary health care and not from a specific study, such as an RCT. This means that the participants were free to perform the test on their own initiative while waiting for their appointment, since the computers were freely accessible in or near the waiting room. In addition, as part of the established daily routine, the PHC staff were asked to refer patients to the test after the consultation whenever staff thought that this was appropriate. This invitation mechanism was similar in all the PHC centres, and the individual recruitment to the test by staff or through the patient's own initiative were ongoing at the same time throughout the whole study period.

After completing an e-SBI but before getting a personalised printout, each of the participants was asked if he or she would agree to participate in a followup mail survey three months later. Participants accepting the invitation were asked to register their national identification number at the end of the test. 

In the analyses in this study the patients were divided into two groups. The first group consisted of patients who performed the e-SBI on their own initiative—this group is called the *self-referred group* throughout this paper. The second group of patients was invited to perform the e-SBIs after their appointments with the PHC staff—this group is called the *staff-referred group* throughout this paper.

Patients in both the self-referred and the staff-referred groups were subcategorised into three types of responder groups: *nonparticipants who *completed the e-SBI but then did not agree to followup surveys; *nonresponders who *completed the e-SBI and agreed to be followed up but did not respond to the followup questionnaire; and *responders* who participated in both the initial and followup measurements. Baseline data for all groups were compared concerning representativity of patients of the included populations in the three-month followup. Comparisons were then made between the staff-referred group and the self-referred group with regards to changes in alcohol consumption at the three month followup.

### 2.4. Followup Measurements

The followup questionnaire was mailed to respondents who had registered their national identification numbers and thereby agreed to be followed up 3 months after their e-SBI was conducted. Addresses for those who agreed to the followup were collected from the Swedish population register. These respondents were asked the same questions about alcohol consumption as in their initial PHC-based e-SBI. One reminder was sent out two weeks after the first followup questionnaire to those who had not returned this questionnaire. 

Since the data collection was performed as part of the routine health care and only included the patient's response to a written questionnaire, after informed consent, there was no need for a formal ethical approval at the time of the start of the data collection according to Swedish law. However, since then, in June 2008, the regulations have been changed, due to uncertainty about how to distinguish between routine and research data collection. For new studies in similar data collection methods, an ethical approval would now be required.

### 2.5. Statistical Methods

Data from the initial and followup measurements were extracted from a database to an Excel file and, thereafter, entered into SPSS 18.0 where the statistical analyses were performed. The significance level of this study was set at ≥.05. Pearson's *χ* ^2^-test and Fisher's exact tests were used to analyse the differences in distribution regarding sociodemographic characteristics (gender and age) by type of categories (Tables 1 and 2), and differences in the proportion of changing from risk to no risk (Table 3). Pearson's *χ* ^2^-test was used when more than two groups were involved; otherwise, Fisher's exact tests were used. Differences in continuous variables, for example, average weekly consumption, and interval variables, for example, frequency of HED occasions per month, were tested with one-way ANOVA when differences involved more than two groups; otherwise, *t*-tests were used. Absolute changes in consumption within each feedback condition were tested using paired *t*-tests. In Table 4, Pearson's *χ* ^2^-test and Fisher's exact tests were used to analyse the differences in answers/statement between nonrisky drinkers and risky drinkers.

In Tables 1 and 2, Pearson's *χ* ^2^-test and Fisher's exact tests were used to analyse the differences in distribution regarding gender and age by type of categories. Pearson's *χ* ^2^-test was used when more than two groups were involved, for example, between all three types of categories. Fisher's exact tests were used, for example, when testing differences in distribution regarding gender between categories nonresponders and responders. In Tables 1 and 2, differences in average weekly consumption and frequency of HED occasions per month were tested by one-way ANOVA and *t*-tests. One-way ANOVA was used when testing differences between all three categories, when testing differences between categories, *t*-test were used. In Table 3, *t*-test was used when testing differences in weekly consumption and number of HD occasions per month between the two types of feedback, referred by staff-group and patient initiated test-group. Absolute changes in consumption within each feedback condition were tested using paired *t*-tests. In Table 4, Pearson's *χ* ^2^-test and Fisher's exact tests were used to analyse the differences in answers/statement between nonrisky drinkers and risky drinkers.

## 3. Results

A total of 7863 patients participated in the e-SBI during the two-year study period. The self-referred participants (*n* = 5051, 64%) outnumbered the staff-referred participants (*n* = 2812, 36%). In total, 3169 patients (40%) were risky drinkers with regards to average weekly consumption and/or frequency of HED, 2497 patients (32%) were nonrisky drinkers, and 1960 (25%) were abstainers. Additional 237 patients (3%) stated that their average weekly alcohol consumption was more than 3-times the risky weekly drinking limit in Sweden; these patients were regarded as outliers or as having misuse/dependence, and their results were, therefore, not included in this analysis. 

**Figure 1:**
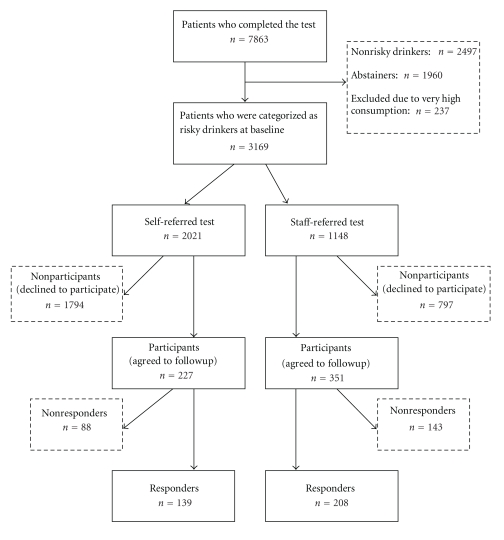
Flowchart of the recruitment of patients as part of the health care routine.

Flowchart of the recruitment of patients as part of the routine health care routine is seen in Figure 1. In the group of risky drinkers, two-thirds were self-referred to the test and one-third was staff-referred to do the test. The proportion of patients agreeing to be followed-up was significantly larger in the staff-referred group than in the self-referred group, 31% versus 11%. Participation data for various groups who took part in the e-SBI are shown in Figure 1. 

### 3.1. Representativity of Participants

Sociodemographic and drinking behaviour characteristics of the risky drinkers who were included in the baseline e-SBI but did not agree to be followed up (nonparticipants), of those who agreed to participate in the followup questionnaire but did not answer the questionnaire (nonresponders), and of those who actually responded (responders) were explored in order to assess the representativity of the patients who responded to the followup questionnaire. The analysis was performed for both the staff-referred and self-referred patients (Tables 1 and 2). 

The staff-referred responders to the followup questionnaire differed from nonresponders to the followup questionnaire, with more women responding to the followup (*P* = .023), but the staff-referred responders did not differ from the nonparticipants with regards to sex (*P* = .637; Table 1). The responders were significantly older than nonresponders *P* = .034 and nonparticipants (*P* ≤ .001). The mean weekly consumption of alcohol measured in grams of alcohol was significantly lower for the responders' group than the nonresponder group (89.7 grams of alcohol per week versus 118.2, *P* = .006) and the nonparticipants group (89.7 grams/week versus 106.6 grams, *P* = .010). There were no significant differences in the mean frequency of HED occasions per month comparing the responders, nonresponders, and nonparticipants (4.5 times per month versus 5.1 (*P* = .691) and 4.7 (*P* = .420)).

The self-referred responders who participated in the followup did not differ from nonresponders (*P* = .678) nor from the nonparticipants in the followup (*P* = .929) with regards to sex. The responders were significantly older than nonresponders (*P* < .001) and non participants (*P* < .001; Table 2). The mean weekly consumption was significantly lower for the responders' group than the nonresponder group (89.3 grams of alcohol per week versus 111.0, *P* = .049) and the nonparticipants group (89.3 grams per week versus 110.6 grams, *P* = .002). There were no significant differences in the mean frequency of HED occasions per month when comparing the responders, nonresponders, and nonparticipants (4.8 times per month versus 4.1 (*P* = .442) and 4.8 (*P* = .982)).

### 3.2. Changes in Alcohol Consumption at the 3-Month Followup among Risky Drinkers

Table 3 displays changes in drinking behaviours, comparing both staff-referred and self-referred responders who were risky drinkers at baseline. Staff-referred responders demonstrated reduced weekly alcohol consumption with an average decrease of 8.4 grams (*P* value for this within-group change after three months =.043). In contrast, self-referred responders showed a nonsignificant increase in weekly alcohol consumption of 2.4 grams (*P* value for this within-group change after three months =.642). The difference in the changes in average weekly consumption between the staff-referred group and the self-referred group was nonsignificant (*P* = .102; Table 3). Further analysis of both groups of participants (*n* = 334) revealed that 40% of all responders decreased their average weekly consumption (−51.9 g/week in average), 21% had an unchanged weekly consumption, and 39% increased their average weekly consumption (42.4 g/week in average). 

Staff-referred responders showed a 49% reduction in the average number of HED occasions per month. The corresponding figure for self-referred responders was a 62% reduction. The within-group reduction with regards to changes in absolute numbers of HED occasions was significant at *P* < .001 for both groups. The difference in the absolute number of HED occasions between the self-referred and staff-referred responder groups was not statistically significant (*P* = .465; Table 3).

About 42% of the staff-referred responders changed their drinking status from a risky drinking levels to a nonrisky drinking levels at 3 months as measured by changes in the study's composite definition of risky drinking, including both the average weekly consumption and/or frequency of HED occasions (Table 3). For the self-referred group, the corresponding proportion was 35%. The difference in the numbers of people in staff- and self-referred groups who moved from risky drinking to nonrisky drinking levels, comparing staff-referred and self-referred respondents, was not statistically significant (*P* = .095). Changes in risky-drinking status in both groups were mainly due to reductions in the frequency of HED. Separate analysis of 203 individuals, from both the staff-referred and self-referred group (with a total of 335 patients), who decreased their number of HED occasions, revealed a corresponding change in the average weekly consumption from 82.5 g/week to 72.7 g/week, in contrast to the more or less unchanged average weekly consumption when considering all participants. 

Separate analyses of men and women revealed no significant difference between sex, with 49% of the staff-referred women (*n* = 85) and 42% of the self-referred women (*n* = 55) regarded as nonrisky drinkers at the 3-month followup (*P* = .338). For men, similar nonsignificant reductions were seen for 37% (*n* = 123) of staff-referred risky drinkers and 31% (*n* = 84) of the self-referred risky drinkers at 3-month followup (*P* = .321).

### 3.3. Changes from Nonrisky Drinker Status to Risky Drinker Status

In order to evaluate the net change in drinking at the 3-month followup among all respondents, an analysis of changes in drinking behaviours described by the patients who were nonrisky drinkers at baseline was also performed. This was possible since all patients were asked to participate in the 3 month followup with no regards to their drinking status at baseline. At the three-month followup, 13% (*n* = 270) of the staff-referred responders who were nonrisky drinkers at baseline and 15% (*n* = 177) of the self-referred responders who were nonrisky drinkers at baseline had increased their drinking to a risky level.

### 3.4. Patients' Perception of the Usefulness of Computerized Advice

Table 4 compares staff-referred and self-referred risky drinkers regarding their perception of the usefulness of the computerized advice. No significant differences were seen in these two patient groups for any of the items. More than 80% had read their personalized advice, remembered that advice, and though that the advice was relevant. Approximately half of the participants had discussed the information with a friend or relative. In the staff-referral group, 26% had discussed their advice with someone at the PHC whereas only 7% in the self-referred group had discussed their advice with PHCS staff (*P* = .087). Very few patients thought the information was difficult to understand.

The patients (*n* = 107) who stated they had decreased their alcohol consumption at the 3-month followup were asked to evaluate the importance of the computerized advice. In the staff-referred group (*n* = 63), 39% stated that the advice had been of great importance or rather great importance compared to 40% in the self-referred group (*P* = .585).

## 4. Discussion

This study assessed changes in drinking behaviours over time in two groups of patients who were attending PHC settings; those who were staff referred (*n* = 208) and those who were self-referred (*n* = 139) to a computerized screening and brief alcohol intervention using a stand-alone computer in the PHC facility. 

At the time of followup, there was no significant difference in reduction in the weekly consumption when comparing the two groups of patients. Both groups of participants reported a fairly low average weekly consumption of alcohol when compared to other international populations described in studies on risky drinking and SBI [2]. This lends additional support to the premise that risky drinking in Sweden is mostly due to frequent occasions of heavy episodic drinking (HED) rather than high average weekly alcohol consumption. 

On the other hand, both groups demonstrated a substantial reduction in the average number of HED occasions. This meant, in many cases, the respondents were no longer regarded as risky drinkers at the 3 month followup. This relatively large shift in reported drinking status occurring in both groups was somewhat surprising when considering previous reports [17–20]. Even when considering the proportion of nonrisky drinkers who changed to risky drinkers at the 3-month followup, the net change from risky to nonrisky drinking status was larger than expected. 

Altogether, there were no significant differences in the reduction in reported alcohol consumption between self-referred and staff-referred patients, suggesting that the staff's referring patients to the test did not have an extra effect on respondent drinking habits. Also, the two groups did not differ in terms of their perception of the usefulness of the computerized advice, as they had, to a similar extent, read the advice, remembered the content, and discussed the information with a friend or relative. These findings imply that making e-SBI available to patients seeking health care does not necessary have to involve the staff. This is a promising result from an implementation viewpoint, since previous research has revealed a reluctance to engage staff in alcohol-preventive measures [3]. 

This study employed the use of touchscreen computers to provide alcohol screening and brief intervention for patients seeking primary health care. The study design applying an e-SBI on site in PHC is a novel one, which has not been replicated in many other studies. While a small number of studies have described the effectiveness of e-SBI in ordinary emergency care [8, 9, 16] or student health care [17, 18], we could find no previous study that had offered e-SBI as part of routine PHC services.

One of the few studies that employs a similar setting to ours, conducted by Kypri et al. [18], was a randomised control trial set in a university health clinic serving young adult students. The Kypri study assessed changes in drinking behaviours in two groups of respondents who were considered risky drinkers. The control group received one electronically delivered BI while the other group of risky drinkers received repeated electronically delivered BIs. While the multidose group showed differences in many alcohol-related measures at the six-month followup, compared to the single dose control group, by the 12-month followup, most of the differences between the groups were nonsignificant. The authors concluded that single-dose e-SBIs delivered in health care settings can be effective in reducing risky drinking. Kypri et al. have also reported an overall effect-size on alcohol consumption on 0.15 in another similar study set in a student health care clinic [19]. This effect-size can be compared to findings in a systematic review of Internet-delivered alcohol interventions where an average effect was calculated to 0.14 using data from 9 studies [10]. Our findings indicate that the effect of our on-site e-SBI appears to be comparable with Internet-delivered interventions.

In another study by Kypri et al., young adult student populations receiving single-dose e-SBIs demonstrated significant differences in alcohol consumption (reductions of 26%) and HED events (reduction of 37%), and they reported fewer personal problems at the six-week followup [19]. These findings are in line with the findings from our study, which demonstrate reductions in alcohol consumption and HED events in the staff-referred group at the 3-month followup. 

A study set in the Netherlands assessed the effectiveness of an e-SBI delivered on the Internet, in a self-selected population of risky drinkers, dividing this population into an intervention group that received an e-SBI and a control group without an intervention [20]. The study found that 17.2% of the intervention group had reduced their drinking to within normal limits, compared to only 5.4% of the control group. This study also points to the general acceptability and usefulness of e-SBIs for adult populations. We note that the magnitude of changes in drinking behaviours, while noticeable in the Netherlands study, is somewhat less than those experienced in our group. It is possible that self-referred Internet-based e-SBI may attract a different kind of population than an e-SBI taking place within a PHC setting. 

Our findings, if replicated in future RCT studies, indicate that e-SBI located on site in PHC settings and made available for self-referred participants has a significant possibility to influence risky drinking. Balanced against the relatively low costs of such interventions, free access to on-site e-SBIs in health care settings may very well provide a cost-effective method by which to assist in behavioural changes concerning alcohol. 

The findings from this study have some limitations, due in large part to the study design, which drew its respondent populations from convenience samples as part of a routine implementation of e-SBI. The lack of a control group means that the findings from the respondent populations can only be compared against themselves and other participants in this same survey. Baseline and followup data from a randomly selected control group of risky drinkers may have provided more information on the relative effectiveness of self- and staff-referred SBI. 

Information on changes in drinking behaviours in a control group of risky drinkers over a three-month period might have also helped us interpret the overall usefulness of e-SBIs in this population, by helping to answer the question whether such sharp reductions in HED occasions, as documented in both respondent groups in this study, are seen in other groups of risky drinkers over a similar period of time, or might the observed change simply be a case of regression to mean? Unfortunately it was not possible for us to perform the study as an RCT with a control group for logistical reason and due to the fact that the e-SBI was set up as part of the daily routine in our health care district. Another reason for the sharp reduction in alcohol consumption could be that we used a paper and pen questionnaire at followup rather than resurveying using an electronic format. However, this has probably not affected the comparison between the two groups of patients; self-referred and staff-referred, since the effect of the change of format would have been the same in both groups.

The participants in this study did not have particularly high average weekly consumption patterns, but rather were more likely to be labelled risky drinkers due to HEDs. This is a drinking pattern that is more prone to natural changes over time. It is possible that this study simply showed a more pronounced regression to the mean over the three-month followup than has been seen in the few studies employing similar methods. Another possible confounding factor to this study could be related to the fact that patients were in busy PHC centres as they participated in e-SBIs. Participants may have downplayed alcohol use, because they thought that the results were somehow being monitored by their PHC providers and they wished to display perceived socially desirable behaviours. However, it would seem plausible that such a social desirability effect would be smaller when using computer technology than face-to-face intervention assessments. Patients who participate in the followup study make a conscious decision to enrol in the study by providing their social security number. This suggests that those who participate are more motivated to be monitored, and, consequently, this could lead to a Hawthorne effect instead of an effect of the intervention as such.

## 5. Conclusions

Our study indicates that e-SBI had the same effect on drinking behaviour in patients who were asked by the staff to perform the test as in patients who did the test on their own initiative without any request from the staff. Balanced against the difficulties to engage primary health care staff in alcohol screening this implies that offering self-referred e-SBI in the waiting room in primary health care settings may very well be a cost-effective method by which to assist in behavioural changes concerning alcohol.

## Figures and Tables

**Table 1:** Comparison of sociodemographic and drinking characteristic between nonparticipant, responders and nonresponders among those patients who were *referred to the test by the staff. *

	Non-participants	*P*-value (nonparticipants versus nonresponders)	Nonresponders	*P*-value (nonresponders versus responders)	Responders	*P*-value (nonparticipants versus responders)
Gender (*P* = .006)	*n* (%)		*n* (%)		*n* (%)	
Male	455 (57)		102 (71)		123 (59)	
Female	342 (43)		41 (29)		85 (41)	
Total	797 (100)	.002	143 (100)	.023	208 (100)	.637

Age (*P* = .000)	*n* (%)		*n* (%)		*n* (%)	
18–20	66 (8)		9 (6)		5 (2)	
21–30	110 (14)		16 (11)		10 (5)	
31–40	84 (11)		13 (9)		23 (11)	
41–50	126 (16)		24 (17)		31 (15)	
51–60	188 (24)		38 (27)		52 (25)	
≥61	215 (27)		42 (30)		87 (42)	
Total	789 (100)	.816	142 (100)	.034	208 (100)	.000

Weekly consumption, g/week (*P* = .010)						
Median (range)	72 (480)		96 (480)		72 (468)	
Mean (SE)	106.6 (3.2)	.170	118.2 (8.5)	.006	89.7 (5.7)	.010

Frequency of HED, no. of HED occasions/month (*P* = .712)						
Median (range)	3 (30)		3 (30)		3 (30)	
Mean (SE)	4.7 (0.2)	.522	5.1 (0.6)	.691	4.5 (0.4)	.420

**Table 2:** Comparison of sociodemographic and drinking characteristic between nonparticipant, responders and nonresponders among those patients who did the test *on their own initiative. *

	Non-participants	*P*-value (nonparticipants versus nonresponders)	Nonresponders	*P*-value (non-responders versus responders)	Responders	*P*-value (nonparticipants versus responders)
Gender (*P* = .839)	*n* (%)		*n* (%)		*n* (%)	
Male	1074 (60)		50 (57)		84 (60)	
Female	720 (40)		38 (43)		55 (40)	
Total	1794 (100)	.579	88 (100)	.678	139 (100)	.929

Age (*P* = .000)	*n* (%)		*n* (%)		*n* (%)	
18–20	187 (11)		15 (17)		3 (2)	
21–30	389 (22)		24 (27)		15 (11)	
31–40	338 (19)		16 (18)		19 (14)	
41–50	312 (18)		13 (15)		21 (15)	
51–60	255 (14)		9 (10)		30 (22)	
≥61	303 (17)		11 (13)		51 (37)	
Total	1784 (100)	.232	88 (100)	.000	139 (100)	.000

Weekly consumption, g/week (*P* = .031)						
Median (range)	84 (504)		90 (432)		72 (420)	
Mean (SE)	110.6 (2.2)	.965	111.0 (9.5)	.049	89.3 (6.4)	.002

Frequency of HED, no. of HED occasions/month (*P* = .654)						
Median (range)	3 (30)		3 (29)		3 (30)	
Mean (SE)	4.8 (0.2)	.351	4.1 (0.6)	.442	4.8 (0.6)	.982

**Table 3:** Changes in drinking variables between baseline and at 3 month followup among staff-referred and self-referred patients.

	Staff-referred group *n* = 208	Self-referred group *n* = 139	*P*-value
*Average weekly consumption *(g)	*n* = 203	*n* = 131	
Baseline, mean (median)	91.0 (72)	88.2 (72)	.757
Followup, mean (median)	82.6 (60)	90.6 (72)	.317
Absolute change (*P*-value)	−8.4 (0.043)^a^	2.4 (0.642)^b^	.102
Relative change (%)	−9	2	

*Number of HED occasions per month *	*n* = 199	*n* = 136	
Baseline, mean (median)	4.5 (3)	4.5 (3)	.897
Followup, mean (median)	2.3 (1)	1.7 (1)	.117
Absolute change (*P*-value)	−2.2 (0.000)^c^	−2.8 (0.000)^d^	.465
Relative change (%)	−49	−62	

*Changed from risk to no risk*	*n* = 208	*n* = 139	
Changed from risk to no risk, %	42	35	.095

^a^Test for change in average weekly intake within the “Staff-referred group”.

^b^Test for change in average weekly intake within the “Self-referred group”.

^c^Test for change in number of HED occasions per month within the “Staff-referred” group.

^d^Test for change in number of HED occasions per month within the “Self-referred test” group.

**Table 4:** Staff-referred and self-referred risky drinkers perception of the usefulness of the computerized advice comparing.

	Staff-referred *n* (%)	Self-referred *n* (%)
*Read the written advice about your alcohol habits (0.585)*		
Yes, I read it thoroughly	87 (43)	59 (44)
Yes, but not so thoroughly	84 (41)	59 (44)
No, I did not read it	7 (3)	1 (1)
Did not get a written printout	13 (6)	9 (7)
Do not remember	13 (6)	7 (5)
Total	**204 (100)**	**135 (100)**

*Remembered the content of the advice concerning alcohol habits (P* = .272)		
Yes	132 (77)	84 (71)
No	39 (23)	34 (29)
Total	**171 (100)**	**118 (100)**

*The information was relevant (P* = .037)		
Yes	139 (82)	94 (80)
No	27 (16)	14 (12)
Do not remember	3 (2)	9 (8)
Total	**169 (100)**	**117 (100)**

*Discussed the information about alcohol with a friend or relative (P* = .974)		
Yes	77 (45)	54 (46)
No	86 (50)	58 (50)
Do not remember	8 (5)	5 (4)
Total	**171 (100)**	**117 (100)**

*Discussed the information about alcohol habits with someone at the PHC unit (P* = .087)		
Yes	15 (26)	8 (7)
No	143 (84)	109 (92)
Do not remember	2 (1)	1 (1)
Total	**171 (100)**	**118 (100)**

*The information about alcohol was easy/difficult to understand* (*P* = .056)		
Easy	108 (92)	157 (92)
Difficult	5 (4)	13 (8)
Do not remember	5 (4)	1 (1)
Total	**118 (100)**	**171 (100)**
